# Expression of Concern: Combinatorial approach of *in silico* and *in vitro* evaluation of MLH1 variant associated with lynch syndrome like metastatic colorectal cancer

**DOI:** 10.1042/BSR-20200225_EOC

**Published:** 2020-06-24

**Keywords:** Colorectal cancer, Lynch syndrome, WES, MLH1, In silico tools, In vitro analysis

The Editorial Office has been made aware of potential issues surrounding the scientific validity of this paper, hence has issued an expression of concern to notify readers whilst the Editorial Office investigates.

